# Supplementary material for: Standing genetic variation fuels rapid adaptation to ocean acidification
Source: Nat Commun. 2019 Dec 20;10:5821. doi: 10.1038/s41467-019-13767-1 (PMC6925106; doi:10.1038/s41467-019-13767-1)
Supplement: Supplementary file 1 — Supplementary Information [file 41467_2019_13767_MOESM1_ESM.pdf]

Standing genetic variation fuels rapid adaptation to ocean acidification

Bitter *et al.*

Supplementary Information

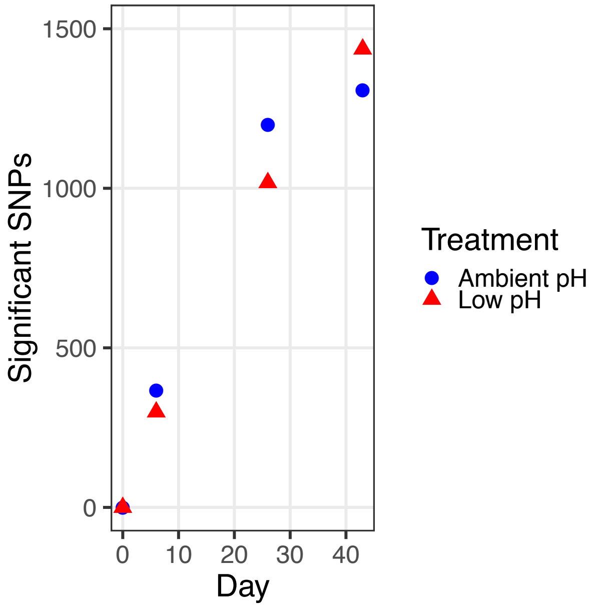

Supplementary Figure 1 | Changes to the number of significant SNPs identified throughout the larval period. Blue circles correspond to ambient and red triangles to low pH treatments) The number of significant SNPs identified reported has been standardized by the number of replicate buckets on each sampling day in order to account for increased power associated with increased replication

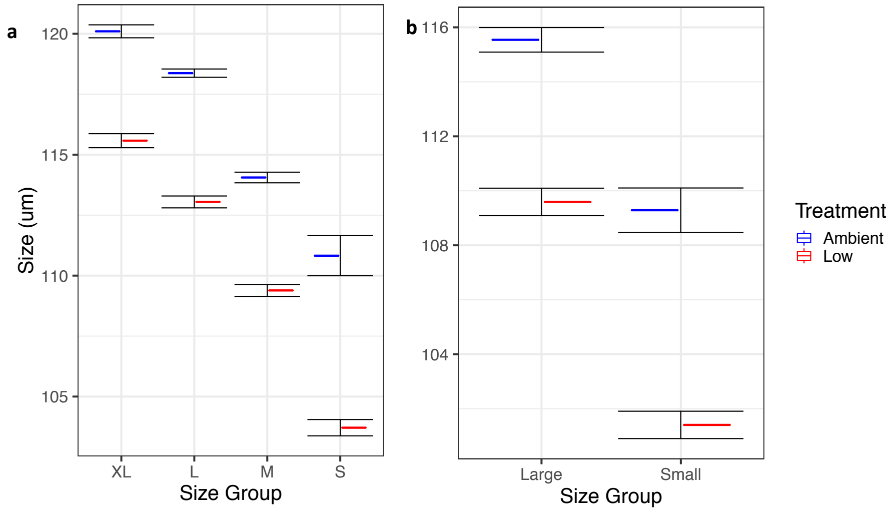

Supplementary Figure 2 | Efficacy of larval size separation method. (a) Pilot data demonstrating the selective isolation of 4 consecutively smaller groups of larvae (mean shell length +/- s.e.) in ambient (blue) and low pH (red) cultured larvae (*N* = 53-324 per size group; XL = extra large; L = large; M = medium; S = small). **(b)** Shell length of larvae (mean +/- SE) in largest (top 20 %) and smallest (bottom 80 %) individuals in ambient (blue) and low pH (red) conditions from present study (*N* = 62 – 177 per size group)

Supplementary Table 1 | Carbonate chemistry for flow-through experimental system used on days 0-26. Four header tanks (two per treatment) each distributed pH adjusted seawater to three replicate buckets. Low/Amb_1/2 correspond to treatment replicates drawing water from separate header tanks, thus one replicate bucket per header tank is represented in the table. Time-series pH and temperature data were generated using in autonomous sensor were generated in each representative replicate bucket, and average values (+/- SD) are presented . Aragonite saturation (Ω_a) and pCO_2 were computed using average pH and AT for each representative replicate. Alkalinity (AT) and salinity samples were generated from discrete samples taken from each of the four header tanks every other day.

pH treatment	pH_T	Ω_a	pCO_2 (µatm)	AT (µmol/kg)	T (°C)	SAL (ppt)
Low_1	7.43 (+/- 0.03)	0.85	2127	2565 (n = 11)	17.3 (+/-0.1)	37.6 (+/-0.1) (n = 11)
Low_2	7.43 (+/- 0.03)	0.85	2130	2569 (n = 11)	17.2 (+/-0.1)	37.6 (+/-0.1) (n = 11)
Amb_1	8.01 (+/- 0.01)	3.32	378	2565 (n = 11)	17.2 (+/-0.1)	37.6 (+/-0.1) (n = 11)
Amb_2	8.01 (+/- 0.01)	3.31	378	2561 (n = 11)	17.2 (+/-0.1)	37.6 (+/-0.1) (n = 11)

Supplementary Table 2 | Carbonate chemistry generated from static cultures used to rear larvae from days 27-43. Low/Ambient_# correspond to each remaining replicate bucket during this portion of the experiment. Discrete measurements of pH, total alkalinity, temperature, and salinity were taken daily and average values (+/- s.d.) are reported. Aragonite saturation (Ω_a) and pCO_2 were computed using average pH and AT for each replicate.

pH Treatment	pH_T	Ω_a	pCO_2 (µatm)	AT (µmol/kg)	T (°C)	SAL (ppt)
Low_1	7.53 (+/-0.10)	1.1	1675	2576 (+/-13)	17.3(+/-0.2)	37.4(+/-0.2)
Low_2	7.53 (+/-0.09)	1.0	1716	2576 (+/-13)	17.3(+/-0.2)	37.4(+/-0.2)
Low_3	7.54 (+/-0.10)	1.1	1633	2576 (+/-13)	17.3(+/-0.2)	37.4(+/-0.2)
Ambient_1	8.08 (+/-0.10)	3.2	403	2579 (+/-13)	17.3(+/-0.2)	37.4(+/-0.2)
Ambient_2	8.01 (+/-0.07)	2.8	488	2579 (+/-13)	17.3(+/-0.2)	37.4(+/-0.2)
Ambient_3	8.00 (+/-0.08)	2.8	501	2579 (+/-13)	17.3(+/-0.2)	37.4(+/-0.2)

Supplementary Table 3 | Changes to F_{ST} across ranges of feasible larval population sizes. F_{ST} was computed between the Day 0 larval population and the larval population in each treatment using *poolFstat*. Rows bolded correspond to input population sizes used for data presented in Figure 3c. Note that in instances where N>1, the average (+/- standard deviation) F_{ST} across all replicate buckets is reported, whereas Figure 3c depicts F_{ST} values from each independent replicate bucket.

Day	*Treatment*	*N*	*n*	*F_{ST} (+/- s.d.)*
6	Ambient	1	10000	0.0066
**6**	**Ambient**	**1**	**25000**	**0.0067**
6	Ambient	1	40000	0.0067
6	Ambient	1	70000	0.0067
6	Ambient	1	90000	0.0067
6	Low	1	10000	0.0062
**6**	**Low**	**1**	**25000**	**0.0062**
6	Low	1	40000	0.0062
6	Low	1	70000	0.0062
6	Low	1	90000	0.0062
25	Ambient	3	1000	0.0084 (+/- 0.0017)
**25**	**Ambient**	**3**	**2500**	**0.0086 (+/- 0.0017)**
25	Ambient	3	4000	0.0087 (+/- 0.0017)
25	Low	3	1000	0.0074 (+/- 0.0008)
**25**	**Low**	**3**	**2500**	**0.0076 (+/- 0.0008)**
25	Low	3	4000	0.0077 (+/- 0.0008)
43	Ambient	2	100	0.0087 (+/- 0.0033)
**43**	**Ambient**	**2**	**250**	**0.010 (+/- 0.0033)**
43	Ambient	2	400	0.011 (+/- 0.0033)
43	Low	3	100	0.0073 (+/- 0.0016)
**43**	**Low**	**3**	**250**	**0.0086 (+/- 0.0016)**
43	Low	3	400	0.0092 (+/- 0.0016)
