# Supplementary material for: Standing genetic variation fuels rapid adaptation to ocean acidification
Source: Nat Commun. 2019 Dec 20;10:5821. doi: 10.1038/s41467-019-13767-1 (PMC6925106; doi:10.1038/s41467-019-13767-1)
Supplement: Supplementary file 3 — Description of Additional Supplementary Files [file 41467_2019_13767_MOESM3_ESM.pdf]

Description of Additional Supplementary Files

File name: Supplementary Data 1
Description: Annotations for ambient, low pH, and shared outlier loci (all contained as separate tabs within file).

File name: Supplementary Data 2
Description: Ambient pH outlier loci .fasta sequences.

File name: Supplementary Data 3
Description: Low pH outlier loci .fasta sequences.

File name: Supplementary Data 4
Description: Shared pH outlier loci .fasta sequences.

File name: Supplementary Data 5
Description: Annotation for genes displaying signatures of selection for shell growth in ambient pH, low pH, and both pH environments ("sharedpH" genes) (all contained as separate tabs within file).

File name: Supplementary Data 6
Description: Ambient pH shell growth genes .fasta sequences.

File name: Supplementary Data 7
Description: Low pH shell growth genes .fasta sequences.

File name: Supplementary Data 8
Description: Shared pH shell growth genes .fasta sequences.
