# Supplementary material for: Standing genetic variation fuels rapid adaptation to ocean acidification
Source: Nat Commun. 2019 Dec 20;10:5821. doi: 10.1038/s41467-019-13767-1 (PMC6925106; doi:10.1038/s41467-019-13767-1)
Supplement: Supplementary file 12 — Reporting Summary [file 41467_2019_13767_MOESM12_ESM.pdf]

nature research

Corresponding author(s): Mark C. Bitter

Last updated by author(s): Sep 19, 2019

Reporting Summary

Nature Research wishes to improve the reproducibility of the work that we publish. This form provides structure for consistency and transparency in reporting. For further information on Nature Research policies, see Authors & Referees and the Editorial Policy Checklist .

Statistics

For all statistical analyses, confirm that the following items are present in the figure legend, table legend, main text, or Methods section.

n/a	Confirmed	
☐	☒	The exact sample size (*n*) for each experimental group/condition, given as a discrete number and unit of measurement
☒	☐	A statement on whether measurements were taken from distinct samples or whether the same sample was measured repeatedly
☐	☒	The statistical test(s) used AND whether they are one- or two-sided<br>*Only common tests should be described solely by name; describe more complex techniques in the Methods section.*
☐	☒	A description of all covariates tested
☐	☒	A description of any assumptions or corrections, such as tests of normality and adjustment for multiple comparisons
☐	☒	A full description of the statistical parameters including central tendency (e.g. means) or other basic estimates (e.g. regression coefficient) AND variation (e.g. standard deviation) or associated estimates of uncertainty (e.g. confidence intervals)
☐	☒	For null hypothesis testing, the test statistic (e.g. *F*, *t*, *r*) with confidence intervals, effect sizes, degrees of freedom and *P* value noted<br>*Give P values as exact values whenever suitable.*
☒	☐	For Bayesian analysis, information on the choice of priors and Markov chain Monte Carlo settings
☒	☐	For hierarchical and complex designs, identification of the appropriate level for tests and full reporting of outcomes
☒	☐	Estimates of effect sizes (e.g. Cohen's *d*, Pearson's *r*), indicating how they were calculated

Our web collection on statistics for biologists contains articles on many of the points above.

Software and code

Policy information about availability of computer code

Data collection	Phenotypic and genetic data were collected by the authors of this study. A published and publicly available transcriptome from Moreira et al. 2015 (doi: 10.1186/s12864-015-1817-5) was used as a reference for read alignment and to identify annotations of outliers and candidate genes.
Data analysis	Software used in this study: PoPoolation2 (10.1371/journal.pone.0015925); Bowtie 2 (10.1038/nmeth.1923); seacarb (https://cran.r-project.org/package=seacarb); qvalue (http://www.bioconductor.org/packages/release/bioc/html/qvalue.html); Trimmomatic (10.1093/bioinformatics/btu170); Genome Analysis Toolkit (GATK; 10.1002/0471250953.bi1110s43); VCFTools (10.1093/bioinformatics/btr330); poolFstat (10.1534/genetics.118.300900)<br>All custom code is available at https://github.com/MarkCBitter

For manuscripts utilizing custom algorithms or software that are central to the research but not yet described in published literature, software must be made available to editors/reviewers. We strongly encourage code deposition in a community repository (e.g. GitHub). See the Nature Research guidelines for submitting code & software for further information.

Data

Policy information about availability of data

All manuscripts must include a data availability statement. This statement should provide the following information, where applicable:
- Accession codes, unique identifiers, or web links for publicly available datasets
- A list of figures that have associated raw data
- A description of any restrictions on data availability

Exome capture sequencing data generated for this study will be available upon publication as .fastq files within an NCBI SRA project. All data used in reported analyses are publicly available at https://github.com/MarkCBitter

Field-specific reporting

Please select the one below that is the best fit for your research. If you are not sure, read the appropriate sections before making your selection.

☐ Life sciences ☐ Behavioural & social sciences ☒ Ecological, evolutionary & environmental sciences

For a reference copy of the document with all sections, see nature.com/documents/nr-reporting-summary-flat.pdf

Ecological, evolutionary & environmental sciences study design

All studies must disclose on these points even when the disclosure is negative.

Study description	We tracked the phenotypic distributions and trajectories of 29,400 single nucleotide polymorphisms (SNPs), from the embryo stage through larval pelagic growth and settlement in a genetically diverse larval population, within an artificially imposed ambient (pHT 8.05) and extreme low pH treatment (pHT 7.4) using the Mediterranean mussel, Mytilus galloprovincialis.
Research sample	Adult M. galloprovincialis were collected to generate gametes and produce a population of genetically diverse larvae, which we tracked throughout development in two pH environments.
Sampling strategy	Logistical constraints set the number of treatment replicates in this experiment (N = 6 per treatment).
Data collection	Phenotypic data was collected by photographing larvae using brightfield microscopy and shell size analysis in imageJ. This was completed by M.C.Bitter.<br>Genetic data was collected by exome capture. Genomic DNA extractions were conducted by M.C.Bitter, library preparations and hybridization of gDNA to capture array was conducted by Arbor Biosciences. Sequencing on Illumina HiSeq4000 was conducted by the University of Chicago Genomics Core Facility.
Timing and spatial scale	The experiment ran from 1 October 2017-13 November 2013. This was the timescale over which the mussel larvae developed from embryos to settled individuals.
Data exclusions	No data were excluded from analysis.
Reproducibility	We used six indepndent replicates per experiment.
Randomization	Larvae in each replicate bucket were seeded from the same, well-mixed starting population.
Blinding	Blinding is not relevant.

Did the study involve field work? ☒ Yes ☐ No

Field work, collection and transport

Field conditions	Adult mussels were collected via snorkel from a standing dock in Thau Lagoon, Séte, France in September 2017. Conditions were calm, skies were grey.
Location	Thau Lagoon, Séte, France (43.415oN, 3.688oE). Adult mussels collected in 0.5 m water.
Access and import/export	Samples collected were exported from France to the United States by ULiSSE CNRS.
Disturbance	Only adult, putatively gravid, mussels were collected in order to limit taking individuals from natural population that were not useful to the study's goals.

Reporting for specific materials, systems and methods

We require information from authors about some types of materials, experimental systems and methods used in many studies. Here, indicate whether each material, system or method listed is relevant to your study. If you are not sure if a list item applies to your research, read the appropriate section before selecting a response.

Materials & experimental systems

n/a	Involved in the study
☒	☐ Antibodies
☒	☐ Eukaryotic cell lines
☒	☐ Palaeontology
☐	☒ Animals and other organisms
☒	☐ Human research participants
☒	☐ Clinical data

Methods

n/a	Involved in the study
☒	☐ ChIP-seq
☒	☐ Flow cytometry
☒	☐ MRI-based neuroimaging

Animals and other organisms

Policy information about studies involving animals; ARRIVE guidelines recommended for reporting animal research

Laboratory animals	N/A
Wild animals	Adult mussels (> 1 year) were removed by hand by dislodging them from the standing dock.<br>After mussels were spawned, they were lethallly dissected for tissue samples for DNA analysis.
Field-collected samples	Mussels were transported from Thau Lagoon to the Laboratoire d'Océanographie (LOV) in Villefranche-sur-Mer, France and stored in a flow-through seawater system maintained at 15.2oC until spawning was induced.
Ethics oversight	No ethical approval was required for this study.

Note that full information on the approval of the study protocol must also be provided in the manuscript.
